# Inhibitory effects on HAV IRES-mediated translation and replication by a combination of amantadine and interferon-alpha

**DOI:** 10.1186/1743-422X-7-212

**Published:** 2010-09-03

**Authors:** Lingli Yang, Tomoko Kiyohara, Tatsuo Kanda, Fumio Imazeki, Keiichi Fujiwara, Verena Gauss-Müller, Koji Ishii, Takaji Wakita, Osamu Yokosuka

**Affiliations:** 1 Department of Medicine and Clinical Oncology, Graduate School of Medicine, Chiba University, 1-8-1 Inohana, Chuo-ku, Chiba 260-8670, Japan; 2 Department of Virology II, National Institute of Infectious Diseases, 4-7-1, Gakuen, Musashi-Murayama, Tokyo 280-0011, Japan; 3 Institute of Medical Molecular Biology, University of Lübeck, Ratzeburger Allee 160, D-23538 Lübeck, Germany; 4 Department of Dermatology, Graduate School of Medicine, Osaka University, Osaka 565-0871, Japan

## Abstract

Hepatitis A virus (HAV) causes acute hepatitis and sometimes leads to fulminant hepatitis. Amantadine is a tricyclic symmetric amine that inhibits the replication of many DNA and RNA viruses. Amantadine was reported to suppress HAV replication, and the efficacy of amantadine was exhibited in its inhibition of the internal ribosomal entry site (IRES) activities of HAV. Interferon (IFN) also has an antiviral effect through the induction of IFN stimulated genes (ISG) and the degradation of viral RNA. To explore the mechanism of the suppression of HAV replication, we examined the effects of the combination of amantadine and IFN-alpha on HAV IRES-mediated translation, HAV replicon replication in human hepatoma cell lines, and HAV KRM003 genotype IIIB strain replication in African green monkey kidney cell GL37. IFN-alpha seems to have no additive effect on HAV IRES-mediated translation inhibition by amantadine. However, suppressions of HAV replicon and HAV replication were stronger with the combination than with amantadine alone. In conclusion, amantadine, in combination of IFN-alpha, might have a beneficial effect in some patients with acute hepatitis A.

## Short report

Hepatitis A virus (HAV), a member of the family Picornaviridae, causes acute hepatitis and occasionally fulminant hepatitis, a life-threatening disease. As the broad epidemiological picture of hepatitis A changes, the public health importance of this disease is being increasingly recognized [1]. It is a significant cause of morbidity worldwide, although the mortality rate due to hepatitis A is low (improved intensive care and transplantation have contributed to a reduction in deaths). Improved sanitation and living standards mean that fewer countries remain highly endemic, but the risk of HAV infection is present in countries lacking HAV immunity or where the endemicity of hepatitis A is low or intermediate [1]. In such situations, these outbreaks can prove to be long and difficult to control. Vaccination and informing the general public about good hygienic measures are important for the prevention of HAV infection, but new therapeutic options are also desirable.

Amantadine, a tricyclic symmetric amine, inhibits HAV replication *in vitro *[2]. We previously reported that amantadine inhibits hepatitis A virus internal ribosomal entry site (IRES)-mediated translation in human hepatoma cells [2]. Interferons (IFNs) also exhibit antiviral effects against HAV infection [2,3]. In the present study, we examined the effects of amantadine with or without IFN-alpha, on HAV IRES activities, HAV subgenomic replicon replication and HAV replication *in vitro *as a proof of concept for the development of a more effective treatment to control HAV infection.

**Figure 1:**
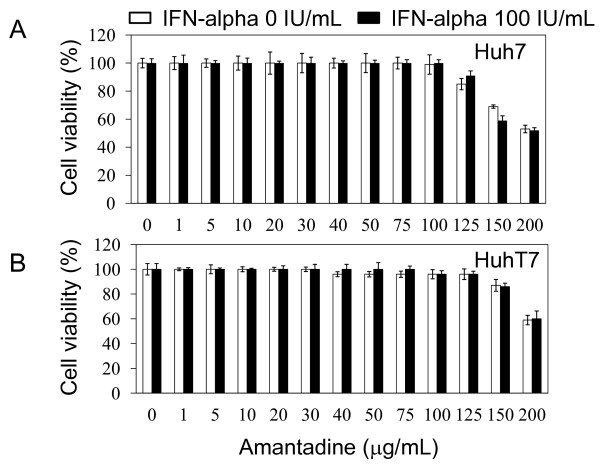
**Effects of amantadine on cell growth and viability**. MTS assays of cells 12 h after treatment with amantadine with or without 100 U/mL interferon (IFN)-alpha. (A) Huh7 cells. (B) HuhT7 cells. Data are expressed as mean ± SD.

First, we evaluated the cytotoxicity of amantadine and IFN-alpha by 3-(4,5-dimethylthiazol-2-yl)-5-(3-carboxymethoxyphenyl)-2-(4-sulfophenyl)-2H-tetrazolium, inner salt (MTS) assay. Amantadine concentrations in a range of 1 - 125 μg/mL and those of 1 - 150 μg/mL for 12-h incubation were non-toxic for Huh7 cells and for HuhT7 cells, respectively (Figures 1A and 1B). Amantadine could be incubated for a short time, e.g., 12 h, with the cells, and then the dose of amantadine could be increased to higher than 100 μg/mL. With the combination of amantadine and 100 IU/mL IFN-alpha, we did not observe increased cytotoxicity compared with amantadine alone.

**Figure 2:**
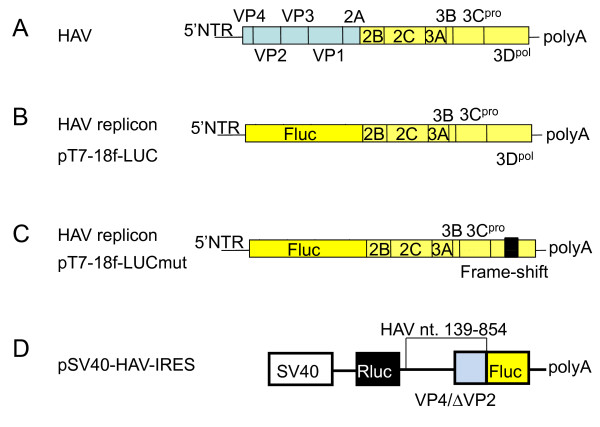
**Structures of reporter constructs used in this study. (A) **Structure of HAV genome. **(B) **Structure of the replication-competent HAV replicon (HAV replicon) pT7-18f-LUC, which contains an open-reading frame of firefly luciferase (Fluc) flanked by the first four amino acids of HAV polyprotein and by 12 C-terminal amino acids of VP1. This segment is followed by P2 and P3 domains of HAV polyprotein (HAV strain HM175 18f) [9,10]. **(C) **Structure of replication-incompetent HAV replicon (mut) (mut-HAV replicon) pT7-18f-LUCmut, which contains a frame-shift mutation in the polymerase 3 D [9,10]. **(D) **Bicistronic reporter constructs: pSV40-HAV IRES was described previously [2,4]. It encodes the Renilla luciferase genes (Rluc), the internal ribosomal entry site (IRES) HAV HM175, and the firefly luciferase gene (Fluc) under the control of the simian virus 40 promoter (SV40).

**Figure 3:**
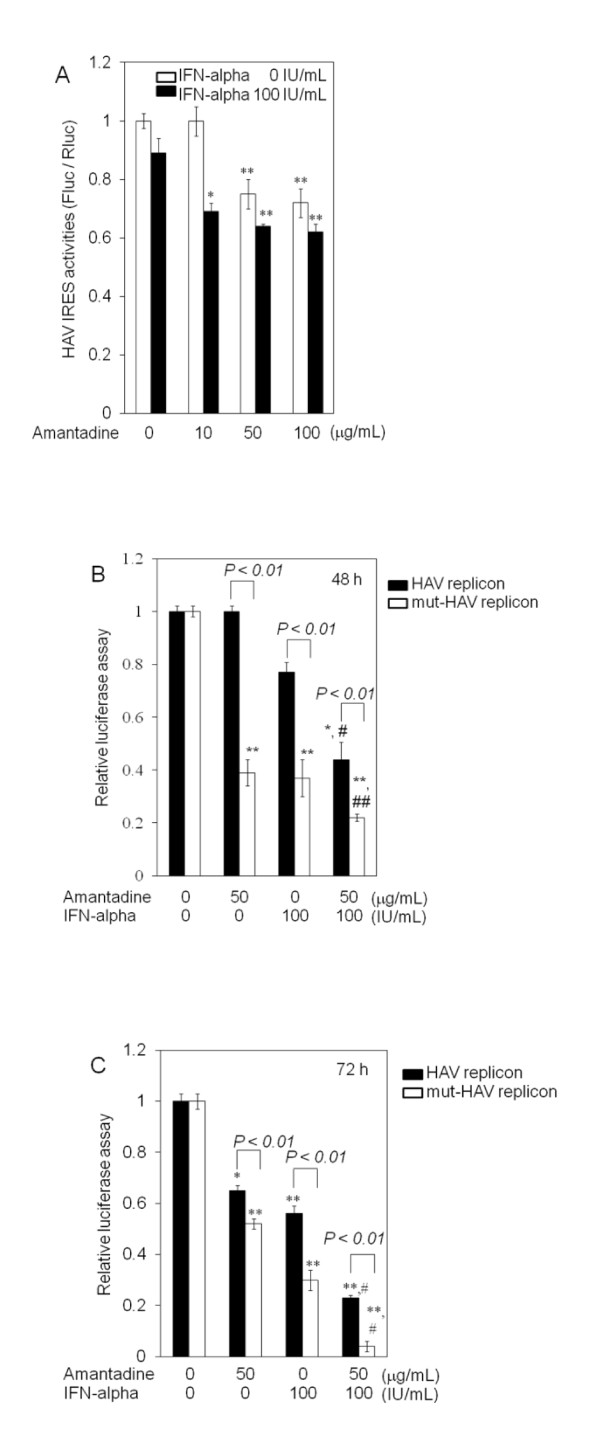
**(A) Effects of amantadine with or without interferon on the hepatitis A virus (HAV) internal ribosomal entry site (IRES) activities in Huh7 cells**. Approximately 2 × 10^5 ^cells were seeded on a 6-well tissue culture plate (Iwaki Glass, Tokyo, Japan) 24 h prior to transfection. pSV40-HAV-IRES (0.3 μg) was transfected into Huh7 cells using the Effectene transfection reagent (Qiagen, Tokyo, Japan). 24 h after transfection, amantadine and/or IFN in various concentrations was added to cells. 48 h after transfection, cell extracts were prepared, and luciferase assays were performed using the Dual Luciferase assay system (Toyo Ink, Tokyo, Japan) according to the manufacturer's instructions [2]. For controlling the variations in transcription, IRES activity was assessed by measuring the ratio of *Renilla *and firefly luciferases. All samples were run in triplicate. *Renilla *and firefly luciferase activities were measured as relative light units using a luminescencer (JNRII-AB-2300; ATTO, Tokyo, Japan). **(B, C) Effects of amantadine with or without interferon on the HAV subgenomic replicon replication in HuhT7 cells. (B) **48 h after transfection and **(C) **72 h after transfection. Black columns, replication-competent HAV replicon; white columns, replication-incompetent HAV replicon (mut). Relative luciferase activities without any treatments were set at 1. Data are expressed as mean (columns) ± SD (vertical lines). *P < 0.05 and **P < 0.01, compared with untreated control by Student's t test. #P < 0.01 and ## *P *< 0.05, compared with amantadine alone or IFN-alpha alone by Student's t test.

We previously reported that the introduction of siRNA targeted against the 5'NTR region of HAV HM175 inhibits HAV IRES-mediated translation and HAV replication [4]. Interestingly, amantadine and IFN also inhibited HAV IRES-mediated translation and HAV replication [2,3,5-8]. Accordingly, we planned to identify more effective strategies for suppressing HAV IRES-mediated translation and HAV replication. IRES is an attractive target for antivirals because HAV IRES is located in the 5'NTR region, the most conserved region among HAV strains. In the present study, we evaluated the HAV antiviral activity of amantadine and IFN-alpha. We initially examined the effects of this combination on HAV IRES-mediated translation using a luciferase reporter assay. Huh7 cells were transfected with pSV40-HAV IRES reporter vector, encoding SV40 promoter driven-*Renilla reniformis *and firefly luciferase, separated by HAV-IRES (Figure 2) [2], and treated with amantadine and/or IFN-alpha. Inhibition of luciferase activity at different levels was observed with amantadine with or without 100 IU/mL IFN-alpha (Figure 3A). Although the strongest suppression was noted with the combination of 10 μg/mL amantadine and 100 IU/mL IFN-alpha, IFN-alpha showed no additive effect on the translation inhibition by 50-100 μg/mL amantadine. This finding prompted us to examine whether IFN-alpha has additive suppression of HAV replicon replication by amantadine. We have reported that RNA replication of HAV can be analyzed in a DNA-based replicon system using HuhT7 cells that stably express T7-RNA polymerase in the cytoplasm (Figure 1) [9-11]. The luciferase activities determined after transfection of replicon DNA are a direct measure of RNA translation and replication. This is because replication in positive-stranded RNA viruses can be easily assessed with a viral replicon carrying the luciferase gene in place of viral structural genes. Moreover, luciferase activity due to translation or translation and replication can be evaluated when the transfection of a replication-competent replicon (HAV replicon) is compared with that of a replication-incompetent replicon (mut) (mut-HAV replicon) [8].

To further determine the effects of the combination of amantadine and IFN-alpha on HAV replication, we transfected the HAV replicon or mut-HAV replicon into HuhT7 cells, and the drugs were added 24 h later. Reporter assays were performed 48 or 72 h after transfection. The transfection efficacy of HAV replicon was estimated as 20-30% in our systems. Luciferase activity was normalized with respect to the protein concentration of cell lysates. In this DNA-based system, 48 h after transfection, the replication rates of the HAV replicon were 100%, 77%, and 44% compared to those of control when treated with amantadine alone, IFN alone, and their combination, respectively (Figure 3B). On the other hand, since the mut-HAV replicon cannot replicate, the luciferase activity (39%, 37%, and 22% compared to those of control for the same test conditions, respectively) is due to translation of the viral RNA and not replication. Amantadine alone showed 52% at 72 h, higher than 37% at 48 h, supporting the notion that amantadine might suppress translation of the viral RNA. Suppression effects of these treatments were stronger in the mut-HAV replicon than in the HAV replicon. These findings support our observation of the suppression of HAV IRES-mediated translation by amantadine and IFN-alpha. Suppression effects at 48 h after transfection by the combination of amantadine and IFN-alpha against HAV replication were stronger than those by amantadine or IFN-alpha monotreatment. IFN-alpha was more effective than amantadine against the HAV replicon (*P *= 0.0027) (Figure 3B).

Seventy-two hours after transfection, the replication rates of the HAV replicon were 65%, 56%, and 23% compared to those of control when treated with amantadine alone, IFN-alpha alone, and their combination, respectively (Figure 3C). The replication rates of the mut-HAV replicon were 52%, 30%, and 4% of those of control, respectively. IFN-alpha was more effective than amantadine against the replication of HAV replicon or mut-HAV replicon (*P *< 0.001 or *P *< 0.001). Suppression effects of the combination of amantadine and IFN-alpha at 72 h post-transfection were stronger than those of amantadine or IFN-alpha monotreatment. Suppression effects of these treatments were stronger in the mut-HAV replicon than in the HAV replicon. Moreover, it is important to note that the effects of this combination were observed at earlier time points (Figure 3C).

**Figure 4:**
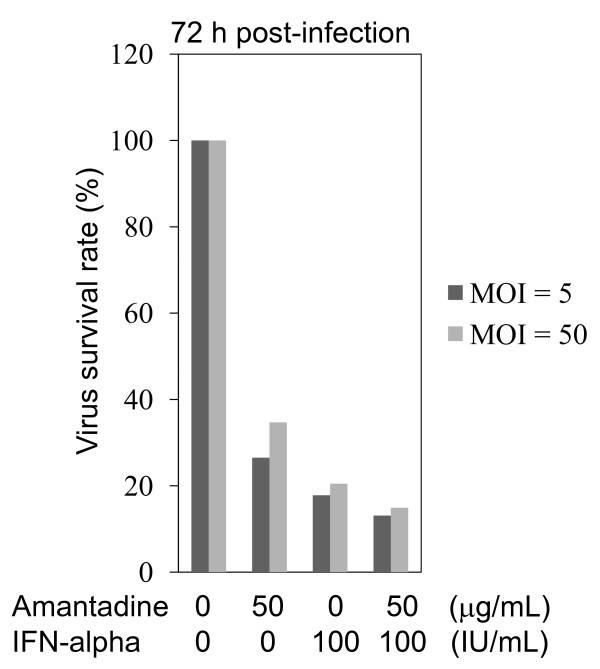
**Effects of amantadine with or without interferon on HAV KRM003 genotype IIIB strain replication in African green monkey kidney cell GL37**. GL37 cell monolayers in 96-well culture plates were infected with HAV [at a multiplicity of infection (MOI) of 5 or 40] for 1 h at 37°C in a CO_2 _incubator. Amantadine and/or IFN was added to cells. After the incubation for 72 h, infected cells were evaluated with ELISA. The rate of virus survival was measured using this equation: Virus survival rate (%) = 100 × Absorbance with drug/Absorbance without drug.

Next, we performed an infectivity assay using the virus to investigate the effects of combination of amantadine and IFN-alpha on tissue culture-adapted HAV strain KRM003 (genotype IIIB, accession no. L20536) propagation in African green monkey kidney GL37 cells [12-14]. GL37 cell monolayers in 96-well culture plates were infected with HAV at a multiplicity of infection (MOI) of 5 or 50 for 1 h at 37°C in a CO_2 _incubator. Without removing the inoculum, drug-containing media were added to appropriate wells. The final concentrations of amantadine, IFN-alpha, and their combination were 50 μg/ml, 100 IU/ml and 50 μg/ml of amantadine and 100 IU/ml of IFN-alpha, respectively. After incubation for 72 h, infected cells were evaluated with ELISA. Suppression of HAV replication by the combination of amantadine and IFN-alpha was stronger than those of amantadine alone, IFN-alpha alone, and untreated control (Figure 4).

IFNs are proteins induced by lymphocytes and other cells including hepatocytes in response to viruses such as HAV. In virus-infected cells, dsRNA activates antiviral interferon pathways and the production of IFN type I. The secreted IFN type I induces a positive feedback loop that results in the expression of interferon-stimulated genes (ISGs), including RNase L and protein kinase R (PKR) [15]. Our study supports the fact that the administration of IFN-alpha suppresses HAV replication through HAV IRES mediated-translation and other mechanisms and that, on the other hand, amantadine suppresses HAV replication mainly through HAV IRES mediated-translation.

There are several reports concerning HAV suppressing intracellular dsRNA-induced retinoic acid-inducible gene I (RIG-I)-mediated IFN regulatory factor 3 (IRF-3) activation to block induction of IFN [16,17]. Yang et al. reported that HAV proteins interact with mitochondrial antiviral signaling protein, an essential component of virus-activated signaling pathways that induce protective IFN responses [18]. However, in this study, the administration of exogenous IFN-alpha could suppress HAV replication, although endogenous IFNs produced by cells also may play an important role in inhibiting viral replication. Further studies will be needed.

Amantadine inhibits the replication of many DNA and RNA viruses and is also used as a drug for the treatment of Parkinson's disease [2]. It is known that the M2 protein of influenza A virus is a target of amantadine [19]. Furthermore, it has been reported to inhibit HAV IRES-mediated translation and replication by our group and other researchers [2,3,5-8].

Therefore, we examined the possibilities of the combination of amantadine and IFN-alpha against HAV because these two drugs were previously reported to be effective against HAV [2,3,5-8]. To our knowledge, this is the first study demonstrating that a combination of amantadine and IFN-alpha can suppress HAV replication more effectively than amantadine or IFN-alpha alone.

## Abbreviations

**HAV**: hepatitis A virus; **IRES**: internal ribosomal entry site; **IFN**: interferon; **MTS**: 3-(4,5-dimethylthiazol-2-yl)-5-(3-carboxymethoxyphenyl)-2-(4-sulfophenyl)-2H-tetrazolium, inner salt.

## Competing interests

The authors declare that they have no competing interests.

## Authors' contributions

LY, TKa, FI and OY conceived and designed the study. LY, TKi and TKa performed the experiments. LY, TKi, TKa and FI analyzed data and wrote the manuscript. TKi, KI and TW contributed to experiments using a whole HAV virus. TKi, TKa and VG contributed to the interpretation of the interpretation of the results and took part to the critical revision of the manuscript. All authors read and approved the final manuscript.
